# Gatifloxacin Loaded Nano Lipid Carriers for the Management of Bacterial Conjunctivitis

**DOI:** 10.3390/antibiotics12081318

**Published:** 2023-08-15

**Authors:** Poorva H. Joshi, Ahmed Adel Ali Youssef, Mihir Ghonge, Corinne Varner, Siddharth Tripathi, Narendar Dudhipala, Soumyajit Majumdar

**Affiliations:** 1 Department of Pharmaceutics and Drug Delivery, School of Pharmacy, University of Mississippi, University, MS 38677, USA; phjoshi@go.olemiss.edu (P.H.J.); aayousse@go.olemiss.edu (A.A.A.Y.); mghonge@go.olemiss.edu (M.G.); cesweene@olemiss.edu (C.V.); narphmreddy@gmail.com (N.D.); 2 Department of Pharmaceutical Technology, Faculty of Pharmacy, Kafrelsheikh University, Kafrelsheikh 33516, Egypt; 3 National Center for Natural Products Research, Research Institute of Pharmaceutical Sciences, University of Mississippi, University, MS 38677, USA; sktripat@olemiss.edu; 4 Research Institute of Pharmaceutical Sciences, University of Mississippi, University, MS 38677, USA

**Keywords:** bacterial conjunctivitis, gatifloxacin, nanostructured lipid carrier, stability, transcorneal, antimicrobial

## Abstract

Bacterial conjunctivitis (BC) entails inflammation of the ocular mucous membrane. Early effective treatment of BC can prevent the spread of the infection to the intraocular tissues, which could lead to bacterial endophthalmitis or serious visual disability. In 2003, gatifloxacin (GTX) eyedrops were introduced as a new broad-spectrum fluoroquinolone to treat BC. Subsequently, GTX use was extended to other ocular bacterial infections. However, due to precorneal loss and poor ocular bioavailability, frequent administration of the commercial eyedrops is necessary, leading to poor patient compliance. Thus, the goal of the current investigation was to formulate GTX in a lipid-based drug delivery system to overcome the challenges with the existing marketed eyedrops and, thus, improve the management of bacterial conjunctivitis. GTX-NLCs and SLNs were formulated with a hot homogenization–probe sonication method. The lead GTX-NLC formulation was characterized and assessed for in vitro drug release, antimicrobial efficacy (against methicillin-resistant *Staphylococcus aureus* and *Pseudomonas aeruginosa*), and ex vivo permeation. The lead formulation exhibited desired physicochemical characteristics, an extended release of GTX over a 12 h period, and was stable over three months at the three storage conditions (refrigerated, room temperature, and accelerated). The transcorneal flux and permeability of GTX from the GTX-NLC formulation were 5.5- and 6.0-fold higher in comparison to the commercial eyedrops and exhibited a similar in vitro antibacterial activity. Therefore, GTX-NLCs could serve as an alternative drug delivery platform to improve treatment outcomes in BC.

## 1. Introduction 

Bacterial eye infections can manifest themselves as blepharitis, conjunctivitis, dacryocystitis, endophthalmitis, keratitis, and orbital cellulitis [1]. Conjunctivitis, inflammation of the conjunctival mucosa, is the most common ocular infection in the United States, with annual incidence rates of approximately 4 million cases and an approximate treatment cost of USD 589 million [2,3]. It is a common complication occurring within an intensive care unit setting, with bacteria contributing to about 50–70% of infectious conjunctivitis [2,4]. If bacterial conjunctivitis (BC) is left untreated, it can affect the adjacent structures and can potentially increase the risk of other extra- or intraocular infections, leading to visual impairment and possibly blindness [4]. 

Fluoroquinolones are considered one of the most effective drug classes for the treatment of ocular bacterial infections, mainly due to their broad spectrum activity and high potency [5]. Moxifloxacin (MOX) and gatifloxacin (GTX) solutions were introduced in 2003 as two new fourth generation fluoroquinolones for the treatment of BC [6]. Recently, their ocular application was extended to cover a wider range of ocular infections, including keratitis and keratoconjunctivitis [7]. These two antibiotics provide better coverage against the leading pathogens in ocular infections, including methicillin-resistant *Staphylococcus aureus* (MRSA) and *Pseudomonas aeruginosa* (*P. aeruginosa*) [8,9]. The Minimum Inhibitory Concentration required to inhibit the growth of 90% of organisms (MIC_90_) against Gram-positive bacteria is significantly lower for fourth generation fluoroquinolones (e.g., GTX and MOX). Furthermore, in the case of Gram-negative bacteria, especially *Pseudomonas aeruginosa*, GTX (MIC_90_ 0.38 μg/mL) was found to be more effective compared to MOX (MIC_90_ 0.75 μg/mL). BC caused by *Pseudomonas aeruginosa* is of particular concern, as it may become more severe and can be a risk factor for keratitis, especially in contact lens wearers [10]. Thus, for this study, GTX was selected for investigation.

GTX is a BCS Class I drug with a high oral bioavailability of 96%. Topical formulations of GTX (as the sesquihydrate) eyedrops (0.5% *w*/*v*) are commercially available as a solution indicated for BC. Frequent administration of aqueous solutions is necessary due to a low residence time on the conjunctival surface [11]. Furthermore, nasolacrimal drainage and rapid tear fluid turnover further contribute to precorneal elimination of the administered dose [12]. As a consequence, only 1–5% of the drug administered topically penetrates through to the intraocular tissues [12,13]. 

Recently, lipid-based drug delivery systems have showcased great potential as controlled drug delivery systems, mainly in terms of enhanced bioavailability, dose reduction, and penetration through various physiological barriers [14,15,16]. These drug delivery systems slowly release the drug by functioning as a reservoir, while also preventing tear washout and maintaining a prolonged drug release [17,18]. Solid lipid nanoparticles (SLNs) possess a solid lipid core accommodating the drug which, when applied topically as a colloidal dispersion, improves the retention time in the cul-de-sac, leading to enhanced ocular bioavailability [19,20]. Furthermore, due to their small size, SLNs do not cause blurred vision or discomfort when administered topically [19]. However, SLNs are shown to expel the drug due to the solidification and gradual crystallization of the solid lipid molecules [21]. Nanostructured lipid carriers (NLCs) contain a binary combination of solid and liquid lipid(s), that helps overcome instabilities associated with SLNs [21]. A colloidal dispersion of NLCs, like the SLNs, improves the ocular bioavailability of the drug by enhancing corneal permeation. Furthermore, the components used are biocompatible and nonirritant, and can be administered safely to ocular tissues, thereby improving patient compliance [15]. Since GTX (as a sesquihydrate) is commercially available at 0.5% *w*/*v*, this concentration was used as the drug load in the formulations. Moreover, lipid nanoparticles such as SLNs and NLCs are made up of lipids/excipients, which are biocompatible and biodegradable, rendering them in vivo compatible [22].

Hence, in the current study, we aimed to develop stable dispersions of GTX-SLNs and GTX-NLCs which exhibit desired physicochemical characteristics, extended release, and improved transmembrane delivery of GTX, via a hot homogenization method.

## 2. Materials and Methods

### 2.1. Materials

GTX (sesquihydrate) was obtained from ThermoFisher Scientific (Hanover Park, IL USA). Glycerin and Tween^®^ 80 were obtained from Spectrum Pharmaceuticals (Henderson, NV, USA). Geleol^TM^ and Precirol^®^ ATO 5 were gifted by Gatefossé. Amicon^®^ ultra centrifugal filter devices, centrifuge tubes (1.5 mL), glass scintillation vials, HPLC vials, Slide-A-Lyzer™ MINI dialysis devices, vitamin E-d-alpha-tocopherol polyethylene glycol 1000 succinate (TPGS), and all other chemicals were obtained from Fisher Scientific Hampton, NH, USA). High-performance liquid chromatography (HPLC) grade solvents were used for analysis. Microbial strains of methicillin-resistant *Staphylococcus aureus* (ATCC 1708) and *Pseudomonas aeruginosa* (BAA-2018) were obtained from the American Type Culture Collection (ATCC, Manassas, VA, USA). Cation-adjusted Mueller Hinton Broth 2 was obtained from MilliporeSigma (St. Louis, MO, USA). Pel-Freez Biologicals (Rogers, AR, USA) supplied whole eyes of albino rabbits (New Zealand) shipped overnight.

### 2.2. Analytical Method

GTX content in all samples was analyzed using a reversed-phase HPLC-UV system consisting of an Alliance Waters e2695 separations module and a Waters 2489 UV/Vis detector (Milford, MA, USA). The mobile phase was made up of 18 mM phosphate buffer containing 0.1% *v*/*v* triethylamine (pH 2.8, adjusted with dilute phosphoric acid) and methanol in a 60:40 *v*/*v* ratio. The mobile phase was pumped isocratically at 1.2 mL/min through a Waters Symmetry^®^ (150 × 4.6 mm, 5 μm) C_18_ column set at 25 °C, with a detection wavelength (λ_max_) set at 294 nm. The samples were analyzed using a Waters chromatography data system coupled with Empower software [23]. The analytical method was linear over a GTX concentration range of 1–100 μg/mL. The validation of the adopted analytical method is included in the Supplementary Materials (Table S1).

### 2.3. Lipid Screening Studies

Various liquid and solid lipids were evaluated for GTX solubility. Nine different liquid lipids (castor oil, sesame oil, mineral oil, oleic acid, isopropyl myristate, Labrafac^®^ Lipophile WL 1349, olive oil, and Miglyol^®^ 829) and nine solid lipids (Softisan 154, Precirol^®^ ATO 5, Compritol^®^ 888 AO, Geleol^™^, Dynasan^™^ 114, Dynasan^™^116, Gelucire^™^ 44/14, Gelucire^™^ 43/01, and Gelucire^™^ 50/13) were evaluated in the solubility screening studies. An accurately weighed amount (10 mg) of GTX was added to each liquid or solid lipid (200 mg) in separate glass vials (3 mL). This mixture was stirred at 2000 rpm under ambient temperature (80 ± 2 °C) for 10 min [23]. After 10 min, stirring was discontinued and the mixtures were removed from the heat. Upon cooling to room temperature (RT), the vials were visually examined for deposits of GTX (yellowish precipitation). The lipids which visually showed no signs of precipitation/deposits were selected for further saturation solubility studies.

### 2.4. Saturation Solubility in Liquid Lipids

Excess GTX (200 mg) was added to the lead liquid lipid (1 mL) in glass vials placed in a reciprocating water bath (Precision^TM^, Waltham, MA, USA) to determine the solubility of the drug in the lipid. The system was maintained at 25 ± 0.5 °C and oscillated at 100 rpm for 48 h. The GTX–lipid combination was then centrifuged for 20 min (Fisher brand™ AccuSpin 17R) at 13,000 rpm. The supernatant was collected then filtered through a 0.22 µm nylon membrane filter, and then quantified for GTX content following proper dilution using HPLC.

### 2.5. Saturation Solubility in Solid Lipids

Excess drug (200 mg) was added to the lead solid lipid (1 g) in glass vials, which were placed inside a reciprocating water bath operating at 100 rpm for 48 h (80 ± 0.5 °C). Samples (10 µL) were removed from the upper layer of the GTX–lipid mixture. The samples were dissolved in methanol (990 µL) and centrifuged for 20 min at 13,000 rpm. The supernatant was collected, then filtered through a 0.22 µm nylon membrane filter, and quantified for GTX content following proper dilution using the HPLC method.

### 2.6. Preparation of GTX-NLC and GTX-SLN Formulations

GTX-SLNs and GTX-NLCs were formulated using the hot homogenization–probe sonication method. The lipid phase in SLNs comprised of GTX in a solid lipid, while the NLCs incorporated GTX in a blend of solid and liquid lipids. The aqueous phase contained Tween^®^ 80 (surfactant), glycerin (tonicity adjustment), and TPGS (permeation enhancer) in Milli-Q water based on previous studies [1]. Both the phases were allowed to reach ambient temperature in a hot water bath (80 ± 2 °C). In a dropwise fashion, the aqueous phase was added to the molten lipid phase under constant stirring (2000 rpm), forming an emulsion. This emulsion was subjected to homogenization at 14,000 rpm at 70 ± 2 °C for 5 min using a T25 digital Ultra-Turrax homogenizer (IKA Works, Inc., Wilmington, NC, USA). Upon cooling to RT, the emulsion was then subjected to probe sonicated (Sonics Vibra-Cell™, Newtown, CT, USA) at 40% amplitude for 10 min using a 3 mm stepped microtip (10 s pulse on; 10 s pulse off), 500 watts power supply, and 115 volts. Placebo formulations (absence of drug) were prepared and visually examined for excipient–excipient compatibility before preparing drug-loaded SLNs and NLCs.

### 2.7. Measurement of Particle Size (PS), Polydispersity Index (PDI), and Zeta Potential (ZP)

SLN and the NLC formulations were analyzed for average PS (d.nm), PDI, and ZP (mV) using Nano ZS Zen3600 Zetasizer (Malvern Panalytical Inc., Westborough, MA, USA) in disposable, clear, micro cuvettes. The formulation (SLN or NLC) was diluted (100 times) with water prior to initiating the measurements (n = 3) at 25 °C. After being evaluated for PS and PDI, the ZP was evaluated by placing these diluted samples in Zetasizer (DTS1070) cells for measurement (n = 3) at 25 °C.

### 2.8. Assay (GTX Content)

GTX content in the GTX-SLN and GTX-NLC formulations was determined by extraction of the drug in methanol. Briefly, the nanodispersion was diluted 100-fold with methanol in a volumetric flask. The mixtures were sonicated for 10 min and then centrifuged at 13,000 rpm at 25 °C for 20 min. The supernatant was analyzed for GTX content using the previously mentioned HPLC method.

### 2.9. Entrapment Efficiency (EE)

The EE (%) of GTX in the nanodispersions was calculated based on the amount of unentrapped drug in the aqueous phase of the prepared formulations. A total of 300 µL of GTX nano formulation was transferred into Amicon^®^ filter devices (pore size 100 kDa) and centrifuged at 13,000 rpm, following which the filtrate was collected and diluted with methanol (10 times), and the amount of GTX content was quantified using the HPLC method. The percentage of GTX entrapped in the lipid phase was calculated using the formula:

$$\%\mathrm{EE}=\left[\frac{\mathrm{Amount}\;\mathrm{of}\;\mathrm{GTX}\;\mathrm{quantified}\;\mathrm{in}\;\mathrm{assay}\;-\;\mathrm{Amount}\;\mathrm{of}\;\mathrm{free}\;\mathrm{GTX}}{\mathrm{The}\;\mathrm{amount}\;\mathrm{of}\;\mathrm{GTX}\;\mathrm{quantified}\;\mathrm{in}\;\mathrm{assay}}\right]\times \;100$$

### 2.10. Measurement of pH and Viscosity

The pH was measured (n = 3) using a Mettler Toledo pH meter equipped with an Inlab^®^ Micro Pro-ISM probe. Prior to the measurement of the samples, the pH meter was calibrated using standardized buffers of known pH.

The viscosity of the lead GTX-NLC was measured using an Ares G2 strain-controlled rotational rheometer (TA instruments, Waters LLC, Newcastle, DE, USA) at 25° C. The rheometer was equipped with a stainless steel cone-plate geometry of 40 mm diameter and a 2° cone angle, having a 0.047 mm truncation gap. The sample was loaded on the platform until it was sandwiched entirely between the geometry and the platform. A flow ramp over a shear rate range of 0.1–100 s^−1^ was used to measure the viscosity of the formulation.

### 2.11. Fourier Transform Infrared Spectroscopy (FTIR)

The infrared spectra of the samples were collected using an Agilent Cary 660 FTIR Spectrometer (650–4000 cm^−1)^. The bench consisted of a MIRacle Attenuated Total Reflection (Pike Technologies) fitted with a single-bounce, diamond-coated ZnSe internal reflection element. Samples of pure GTX, pure lipid excipients, their corresponding physical mixture, placebo formulation, and the lead GTX-NLC were analyzed.

### 2.12. In Vitro Release-Diffusion Studies

A Slide-A-Lyzer™ dialysis device (10 K molecular weight cut-off) shaped in a cup-like design was used to evaluate the release. The lead NLC formulation or control (200 μL) was added to the dialysis device (donor compartment) that was placed at the mouth of the scintillation vial (receiver compartment). The release solution (20 mL) consisted of isotonic phosphate-buffered saline (IPBS, pH 7.4) containing 2.5% *w*/*v* hydroxypropyl beta-cyclodextrin (HPβCD) maintained at 34 ± 0.5 °C. The study was carried out atop a magnetic stirrer (IKA™ RT 10, IKA Works Inc.) under continuous magnetic stirring at 100 rpm. The commercial ophthalmic solution of GTX contains 0.5% (5 mg/mL) equivalent of the active drug (GTX-C) and was used as a control. At scheduled time intervals, samples were removed from the receptor compartment and replenished with an equal amount of fresh media maintained at 34 ± 2 °C. The withdrawn samples were analyzed using the HPLC method mentioned above. All studies were carried out in triplicate. The release profile data were fitted to four mathematical release models using DDSolver for Microsoft Excel (Office365, 2016), to understand the potential drug release mechanism.

### 2.13. Antimicrobial Efficacy

The antimicrobial activity of the lead GTX-NLC formulation was evaluated against *Pseudomonas aeruginosa* (ATCC BAA-2018) and methicillin-resistant *Staphylococcus aureus* (ATCC 1708). An adapted version of the Clinical and Laboratory Standards Institute (CLSI) method was used to perform susceptibility testing. The lead GTX-NLC formulation and its corresponding placebo were diluted 1:2 fold using cation-adjusted Mueller–Hinton medium at pH 7.0. Diluted samples (10 μL) of the lead formulation were transferred to 96 well assay plates (n = 3). Inocula was prepared as per CLSI protocol by correcting the OD_630_ of microbe suspensions in incubation broth. Alamar Blue™ (5% *w*/*v*) was added to both organisms’ plates. GTX-C was included as a positive control in the microbial assays. The optical density was determined at 35 °C before and after incubation for 24 h, using the Bio-Tek plate reader. Minimum Inhibitory Concentrations (MICs), described as the lowest GTX concentration that allows no visual microbial growth, were calculated for the samples.

### 2.14. Transcorneal Permeation Studies

Permeability of GTX from the lead GTX-NLC and control (GTX-C) formulations were evaluated using excised rabbit corneas obtained from Pel-Freez Biologicals^®^. Immediately upon their receipt, the corneas were excised and washed with isotonic phosphate-buffered saline (IPBS). Each excised cornea was securely mounted on a vertical Franz diffusion cell (spherical joint, PermeGear^®^ Inc., Hellertown, PA, USA), with the epithelial side of the cornea positioned towards the donor chamber containing the formulation (200 μL). The receiver chamber was filled with 5 mL of receiver medium consisting of 2.5% HPβCD dissolved in IPBS (pH 7.4) [24]. HPβCD was included to maintain sink conditions, as cyclodextrins are known to enhance the solubility of fluoroquinolones through the formation of inclusion complexes [25,26]. The media was maintained at ocular surface temperature (34 ± 0.5 °C) under continuous stirring [27,28]. Aliquots of 0.5 mL were collected from the receiver chamber at predetermined time points and replenished with an equal amount of the receiver medium maintained at the same temperature. The duration of the study was 3 h. The samples were quantified for GTX content using the HPLC method previously mentioned. The cumulative amount of GTX permeated (*Q_n_*), steady-state flux (*J_ss_*), and permeability coefficient (*P_eff_*) across the excised rabbit corneas were calculated.

*Q_n_* was calculated based on the following formula:

$$Q_{n}{=V}_{r}C_{r(n)}+\overset{x=n}{\underset{x=1}{\sum }}V_{s(x-1)}C_{r(x-1)}$$

where *V_r_* (mL) is the volume present in the receiver compartment; *C_r(n)_* (µg/mL) is the GTX concentration in the receiver compartment at the nth time point; *n* is the time point; and *V_S_* (mL) is the volume of the aliquot withdrawn at the nth time point.

*J_ss_* was calculated using the following formula:

*J_ss_* = (*dQ*/*dt*)/*A*

where (*dQ*/*dt*) is the rate of transcorneal permeation, calculated using the slope of *Q_n_* plotted against time *A* is the effective area of transcorneal permeation (0.64 cm^2^).

(*P_eff_*)—transcorneal permeability coefficient was calculated using the formula:

*P_eff_* = *J_ss_*/*C*_0_

where *C*_0_ is the initial donor concentration for GTX.

### 2.15. Stability Studies

The lead GTX-NLC was investigated at three different storage conditions (4 ± 2.0 (RF), 25 ± 2.0 (RT), and 40 ± 2 °C (accelerated)). Samples kept at 25 and 40 °C were kept inside an 18 L Heratherme™ compact incubator (Thermo Scientific, Waltham, MA, USA). These samples were assessed at predetermined time points for any possible changes in PS, PDI, ZP, pH, GTX content, and EE.

### 2.16. Scanning Transmission Electron Microscopy (STEM)

STEM analysis was performed using a JSM-7200FLV scanning electron microscope (JOEL, Peabody, MA, USA) attached to a STEM detector with a 30 KV accelerating voltage. The samples for imaging were prepped using a negative staining technique. Briefly, 20 µL of the sample was loaded onto a grid plate. The grid was then washed with distilled water and the excess was wiped off. This grid was then placed in the staining solution for about a minute, the excess solution wiped off, and then air dried for a couple of minutes before being viewed under the microscope.

### 2.17. Statistical Analysis

A one-way analysis of variance (ANOVA) was used for the statistical analysis of data (IBM SPSS Statistics 28 software, Armonk, NY, USA). All the results are presented as mean ± standard deviation. The differences were considered significant at a *p*-value less than 0.05 (*p* < 0.05).

## 3. Results and Discussion

### 3.1. Screening of Lipids

The determination of the solubility of the drug in the lipid is critical in the selection of the liquid and solid lipid combinations. Drug entrapment and loading efficiency are directly impacted by the solubility of the GTX in the lipid(s) [29]. Therefore, an initial lipid screening study was conducted to identify liquid lipids and solid lipids, which would be capable of solubilizing GTX without forming aggregates upon cooling. This is a crucial step in the formulation of a stable lipid nano formulation with the maximum/desired drug loading [30]. From the lipid screening studies, two solid lipids (Geleol^™^ and Precirol^®^ ATO 5) and one liquid lipid (oleic acid) were identified as lead candidates (because they did not show any precipitation after cooling) for the lipid nano formulation development (Table 1).

### 3.2. Saturation Solubility Studies

Saturation solubility studies were performed to quantify the amount of GTX soluble in the selected lead solid and liquid lipids. The studies revealed that 155.0 ± 0.3 mg, 80.0 ± 1.3 mg, and 53.0 ± 1.6 mg of GTX dissolves in oleic acid (l.0 mL), Precirol^®^ ATO 5 (1.0 g), and Geleol^™^ (1.0 g), respectively (n = 3).

### 3.3. Preliminary Studies

Placebo SLNs and NLCs containing the selected solid lipids or a combination of the selected liquid and solid lipids, respectively, were prepared and examined for physical incompatibilities, e.g., cracking, creaming, coalescence, or phase separation (Table 2). All Geleol™-based placebo formulations (P5–P8, P11, and P12) revealed an incompatibility between Geleol™ and other formulation excipients, hence formulations containing Geleol™ were not pursued. Both nanocarriers (SLNs and NLCs) were prepared successfully with Precirol^®^ ATO 5 (P1–P4, P9, P10). These placebo formulations were physically stable for three months (last time point tested) and exhibited no visible signs of aggregation or cracking at the three storage conditions tested, namely RF, RT, and accelerated. Based on these preliminary studies, GTX was loaded in nanocarriers containing Precirol^®^ ATO 5.

### 3.4. Preparation of GTX-Loaded SLNs and NLCs

The physicochemical characteristics and visual observations of different GTX-loaded SLNs and NLCs are presented below in Table 3.

A couple of earlier investigations have prepared and evaluated GTX-loaded ophthalmic formulations. Abul et al. prepared SLNs with 2.0% *w*/*v* poloxamer 188, which is 20 times higher than the concentration for topical ocular listed in the FDA inactive ingredient database, which could cause irritation and toxicity [31]. In addition, the authors used sodium taurocholate and ethanol as the ionic surfactant and co-surfactant, respectively, for ocular delivery, which can also lead to severe irritation and toxicity. Duxfield et al. developed GTX-loaded cationic polymeric nanoparticles for topical ocular application. The authors used acetone as an organic solvent during their nanoprecipitation method, and they did not discuss removing the residual solvents within their research [14]. Khurana et al. formulated GTX ocular inserts by the solvent casting method [32]. This, however, raises concerns about potential residual organic solvents, which may be present within the formulation.

In our study, SLNs and NLCs were prepared using hot homogenization coupled with a probe sonication method. Precirol^®^ ATO 5 (solid) and oleic acid (liquid) were used as the lipids, and Tween^®^ 80 was used as a surfactant during the GTX-SLN and GTX-NLC formulation development. The SLN contained only Precirol^®^ ATO 5, while the NLCs comprised of both Precirol^®^ ATO 5 and oleic acid. The highest reported concentrations of Tween^®^ 80 and glycerin in an FDA-approved topical ophthalmic product are 4.0% and 2.25% *w*/*v*, respectively. Hence, in the current study, concentrations up to 4.0% and 2.25% were investigated. TPGS, an FDA-approved adjuvant, possesses activity against free radicals, thereby exerting a protective role on the biological membrane [33]. It has also been incorporated in ocular formulations as a permeation enhancer, stabilizer, and emulsifier, maximizing drug effects while reducing toxicity [33]. TPGS has been reported to act as a P-gp inhibitor, which could potentially improve the absorption of topically administered P-gp substrates like GTX [33,34].

GTX-SLNs were stable for only 15 days at RT (Table 3), as indicated by drug deposition at the bottom of the vial. Drug molecules dissolve in lipids by arranging themselves between the fatty acid chains or glycerides [35]. However, due to the tendency of solid lipids to crystallize over time, SLNs show a high probability of elimination of the dissolved drug during storage [35]. The NLCs, on the other hand, were stable for over 15 days at RT and, therefore, the investigation was continued with GTX-NLCs. While there was no significant (*p* < 0.05) change in the ZP and assay across the various formulations tested, increasing total lipids increased PS and PDI from 229.5 ± 3.2 (F3; 4.0% *w*/*v*) to 266.8 ± 6.4 (F1; 5.0% *w*/*v*) nm, and 0.16 ± 0.06 (F3) to 0.42 ± 0.05 (F1), respectively (Table 3).

The pH of ocular formulations plays a crucial role in developing an effective, non-irritant drug delivery systems [17]. The pH of human tears is between 6.5 and 7.5; however, topical ophthalmic formulations within the pH range of 3.0 to 8.6 are well tolerated, due to the buffering capacity of the tears [36]. All the GTX-SLNS and NLCs formulated were between a pH of 5.62 and 7.17, well within the tolerated pH range of the eye.

EE (%) also increased significantly (*p* < 0.05), from 73.9 ± 3.2 (F3) to 87.3 ± 2.0 (F1), with an increase in total lipids (Table 3). The lipid-to-drug ratio is a crucial factor in NLC drug loading. Changing the lipid/drug weight ratio from 10:1 (F1 and F2) to 8:1 (F3 and F4) decreased the entrapped drug found within the lipid matrix. This outcome could be due to more total lipids being available for encapsulation of the GTX molecules. Moreover, GTX EE within the GTX-NLC increased on increasing the content of liquid lipid, 91.63 ± 1.5 for F2 compared to 87.3 ± 2.0 for F1, and 81.2 ± 1.8 for F4 versus 73.9 ± 3.2 for F3. Increasing the liquid lipid content could add imperfections within the crystallinity of the solid lipid, which could accommodate more drug and, thus, increase drug loading and EE.

We also varied the amount of total lipids in the formulation (4 or 5% *w*/*v*) with different ratios of solid to liquid lipid (1:1 and 3:2). Formulations prepared with 4% *w*/*v* of total lipids (F3 and F4) showed a lower EE (%) compared to formulations prepared with 5% *w*/*v* of total lipids (F1 and F2). When NLC formulations were prepared with a solid-to-liquid lipid ratio of 1:1 (F2 and F4), a smaller particle size was observed in comparison to formulations prepared with 3:2 (F1) and 3:1 (F3). Formulations prepared with 5% *w*/*v* total lipids were observed to have a larger particle size in comparison to formulations prepared with 4% *w*/*v* total lipids with respect to the ratio of solid to liquid lipid. The PDI is also smaller for NLC formulations prepared with 4% *w*/*v* of total lipids compared to 5% *w*/*v* total lipids.

Formulations with a 1:1 ratio of solid to liquid lipids, F2 (5% *w*/*v* of total lipids) and F4 (4% *w*/*v* of total lipids), show a similar pH of around 6.3. When comparing F2 (5% *w*/*v* of total lipids) and F4 (4% *w*/*v* of total lipids) with regard to EE (%), F2 has a higher EE (%) suggesting the increase in lipid content increased the amount of GTX encapsulated. F2 (5% *w*/*v* of total lipids) shows a slightly higher particle size in comparison to F4 (4% *w*/*v* of total lipids), which could be attributed to the increase in the total amount of lipids used.

### 3.5. Effect of Tween^®^ 80 Concentration on GTX-NLCs

Additionally, the effect of varying Tween^®^ 80 concentrations on PS, PDI, ZP, and EE, and the physical stability of GTX-NLC formulations, was investigated (Table 4).

The key role of the surfactant in the NLC formulation is the stabilization of the nanocarriers in the colloidal systems and minimization of PS growth during storage [37]. The effect of increasing Tween^®^ 80 concentrations, 2.0, 3.0, and 4.0% *w*/*v*, on the physicochemical characteristics of GTX-NLCs, based on a 1:1 solid-to-liquid lipid ratio with different total lipid concentrations, is shown in Table 4. When the surfactant concentration increased from 2 to 4%, a significant decrease (*p* < 0.05) in the PS and PDI was observed without affecting the zeta potential. The decrease in PS and PDI with an increase in Tween^®^ 80 concentration can be attributed to the substantial decline in interfacial tension between the lipid matrix and the suspending aqueous phase [37].

Nanocarrier formulations with PDI value ≤ 0.1 indicates uniform dispersion with narrow PS distribution, with 0.1–0.4 indicating moderate dispersed distribution, while PDI value > 0.4 indicates a highly polydisperse system [38,39]. Therefore, the investigated nanocarriers showed good PDI values (0.13 ± 0.02–0.38 ± 0.04). ZP beyond ±30 mV (absolute value) indicates good physical stability for nanoparticulate formulations, and the stability improves with ZP values closer to ±60 mV [40]. The absence of the effect of the surfactant on the ZP is due to the non-ionic character of this surfactant. The assay of all prepared GTX-NLC formulations were between 98.6 ± 2.1–102.3 ± 1.1% of the theoretical value.

The EE (%) values of all prepared NLC formulations were above 70.9 ± 2.5%. The EE (%) of the nanoparticles increased significantly with an increase in the surfactant concentration (*p* < 0.05). This is consistent with earlier reports that show a similar trend in the EE of solid lipid matrices with respect to surfactant concentration [40]. The increased EE is likely due to adequate surfactant molecules being present to surround the lipid, stabilize it, and prevent its coalescence [37]. This phenomenon ensures the retention of the encapsulated drug molecule within the NLC matrix [37,40]. At higher concentrations, an adequate amount of surfactant is available to surround the lipid, resulting in the stabilization of the suspended particles by preventing coalescence. Therefore, high concentrations of Tween^®^ 80 help maintain the encapsulation of the drug within the nano lipid carrier [37]. This was made evident by F6 and F8 formulations, each containing 4% Tween^®^ 80, which showed the highest EEs at 5% and 4% *w*/*v* lipids, respectively.

Cationic polymeric nanoparticles developed by Duxfield et al. were above 400 nm in size, and only encapsulated about 46.0% of GTX [14]. However, in this study, the EE (%) values of all prepared NLC formulations were above 70.9 ± 2.5%. EE (%) values of more than 60% indicate the successful encapsulation of the drug into the lipid particles during the preparation method [41]. Furthermore, the slow release of GTX from these cationic nanoparticles failed to reach effective concentrations necessary to suppress microbial growth [14].

F8 (containing 4.0% total lipids and 4.0% *w*/*v* Tween^®^ 80) exhibited similar physicochemical characteristics to F2 (containing 5.0% total lipids and 3.0% *w*/*v* Tween^®^ 80); however, F8 exhibited smaller particle size and PDI. The smaller particle size exhibited in F8 can be attributed to the increase in Tween^®^ 80 concentration present in the formulation while containing less total lipids present [42,43]. The excess surfactant concentration in F8 could result in the accumulation of excess surfactant molecules on the NLC particles or the formation of micellar particles, which would cause irritation and toxicity to the eye [44,45,46]. While non-ionic surfactants are widely used in ocular nanoparticle formulations, it is suggested to keep the concentration level of surfactants at the lowest possible.

The increase in total lipid content from 4 (F8) to 5% (F2 and F6) increased the amount of oleic acid, which improves the transcorneal permeation [47]. The increase in liquid lipid (oleic acid) improves the solubilization of the drug, which incorporates more drug into the lipid matrix, thereby improving the formulation stability by reducing the particle crystallinity [48]. Thus, formulations F2 and F6 were selected for further evaluation.

### 3.6. In Vitro Drug Release-Diffusion Test

**Figure 1:**
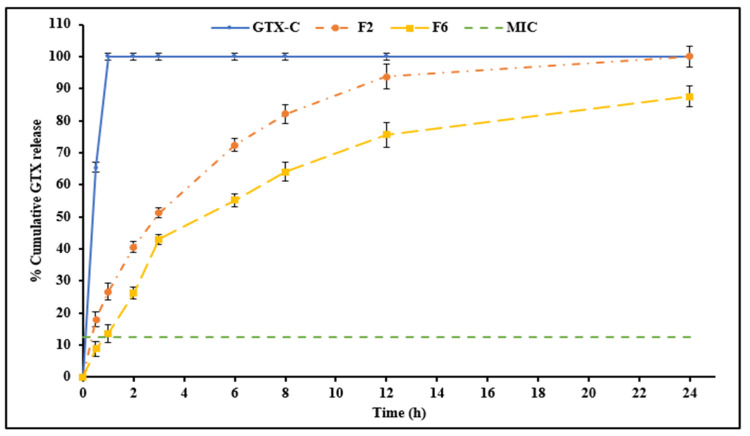
In vitro release profile of gatifloxacin from commercial solution eyedrops and gatifloxacin-containing nanostructured lipid carriers (mean ± SD, n = 3).

The in vitro release-diffusion profiles of GTX from F2, F6, and GTX-C were obtained by the release-diffusion method (data illustrated in Figure 1). The cumulative percentage of GTX released from F2 and F6 were 100.9 ± 1.8% and 92.5 ± 2.2%, respectively, in 24 h. In contrast, GTX-C released 100% GTX within 1 h. Thus, both F2 and F6 NLC formulations extended the release of GTX compared to the commercial GTX-C eyedrops.

The NLCs F2 and F6 released about 41% and 26%, respectively, in 2 h. The extended drug release profiles from the GTX-NLCs is likely due to the slowed release rates of the entrapped GTX from the solid crystalline lipid matrix [49]. However, F2, containing 3% Tween^®^ 80, showed a faster drug release compared to F6, which contained 4% Tween^®^ 80. This could be due to the higher amount of GTX present in the surrounding aqueous phase available to diffuse in case the of F2 (lower EE), as compared to F6 (higher EE). Also, a higher Tween^®^ 80 concentration may lead to a more stabilized crystalline lipid matrix, which could lead to better entrapment of the drug and, thus, slower release [40]. Thus, although higher Tween^®^ 80 concentrations improved EE, it slowed down the rate of GTX release.

Each of the three release models (Korsmeyer–Peppas, Higuchi, and first-order), and regression analysis were carried out for each formulation profile to assess the best fit based on its coefficient of determination (R^2^) [23]. The release model with the highest R^2^ was chosen as the best model to describe release kinetics (Table 5). The release profiles of both F2 and F6 followed the Korsmeyer–Peppas model (based on mathematical model fitting). According to the calculated slope values (n = 0.6), a non-Fickian (0.5 < n < 1.0) GTX release profile, indicating both erosion and diffusion release mechanisms, was suggested. Based on the above data, GTX-NLC formulation F2 was selected for further studies.

### 3.7. Viscosity

Viscosity is a crucial parameter, with values up to 50 cP facilitating easy ocular application of topical products [49]. The mean viscosity of the lead F2 formulation was 11.29 cP between sheer rates of 60–100 s^−1^. Furthermore, formulations with viscosity values towards the lower end are likely to be well-accepted due to minimal blinking pain upon administration [50].

### 3.8. FTIR

**Figure 2:**
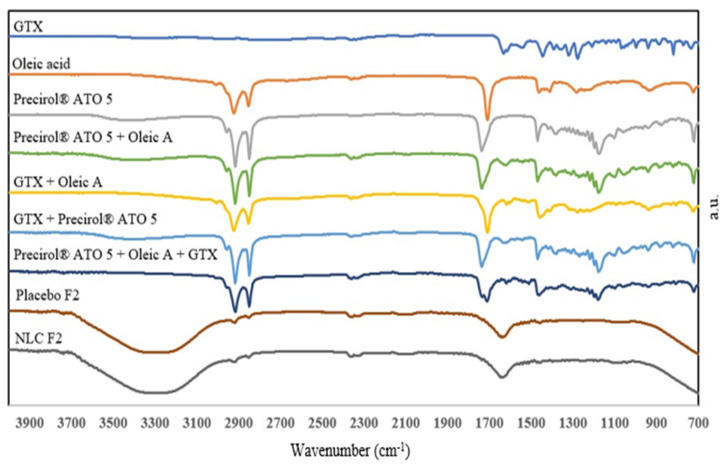
FTIR spectra for GTX, oleic acid, Precirol^®^ ATO 5, GTX-oleic acid, GTX-Precirol^®^ ATO 5, Precirol^®^ ATO 5-oleic acid-GTX, placebo and lead NLC formulations.

The FTIR spectra of GTX, oleic acid, Precirol^®^ ATO 5, physical mixtures of GTX and the excipients, lead NLC formulation (F2) and its corresponding placebo, were collected to identify any interaction between the drug and excipients (Figure 2). GTX peaks were not visible in any of the physical mixture spectra as peaks indicating solubility and entrapment of GTX in the solid–lipid composition [1,51]. The FTIR spectrum of F2 and its placebo possessed a similar broad peak at 3200–3600 cm^−1^ due to O-H bond stretching (aqueous external phase) [12]. Characteristic peaks of the drug were absent in the placebo and the F2 formulations, likely overshadowed by the excipient peaks, indicating no interaction but rather a dispersion of GTX within the lipid matrix.

### 3.9. Transcorneal Study

**Figure 3:**
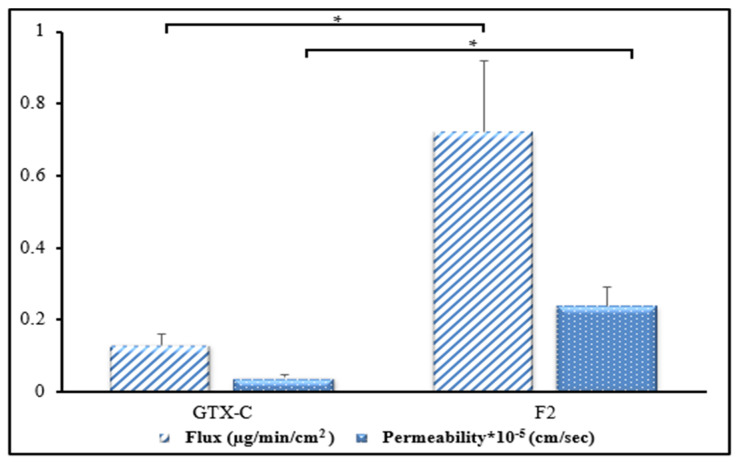
Transcorneal flux and permeability of GTX from commercial solution eyedrops (GTX-C) and lead GTX-NLC formulation (F2) across isolated rabbit cornea (mean ± SD, n = 4). * significantly different at *p* < 0.05.

Abul et al. used goat corneas for their permeation studies, which are less similar to human corneas in comparison to rabbit corneas [52], while Khurana et al. used a Millipore membrane filter to mimic the corneal epithelial barrier, which estimates any improvement in transcorneal permeation difficult [32]. In our study, transcorneal delivery of GTX from the lead NLC formulation was evaluated using rabbit corneas due to their close similarity to human corneas [12]. As shown in Figure 3, the transcorneal flux and permeability of GTX from the F2 were 5.5- and 6-fold higher, respectively, in comparison to GTX-C (control). About 9.16% of the drug permeated across the isolated rabbit cornea within 3 h. NLCs create a film on the corneal surface after topical application [49], wherein the lipid core mimics a drug depot interacting with the tear film’s lipid layer [15]. This property causes the nanocarriers to be retained on the ocular surface [53], thereby increasing ocular bioavailability, while providing sustained release, compared to an aqueous solution which is washed away quickly, thus requiring frequent administration [29]. A receptor-mediated endocytosis uptake mechanism helps internalize lipid particles below 250 nm and across corneal cell membranes, thus enhancing transcorneal delivery [54,55]. The non-ionic Tween^®^ 80 surfactant further improves corneal penetration, allowing the drug to reach the intraocular tissues [56,57].

### 3.10. Stability Studies

**Figure 4:**
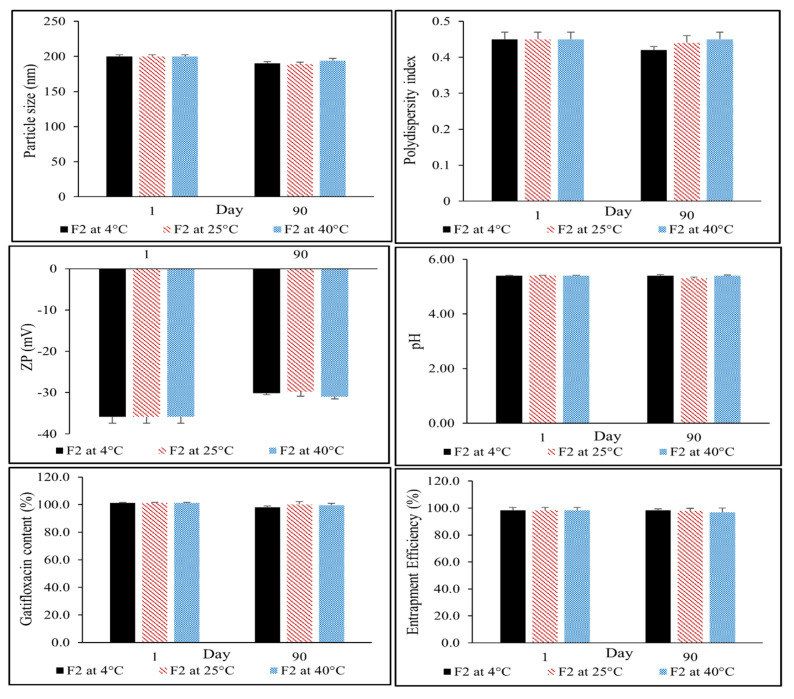
Particle size, polydispersity index, zeta potential (ZP), pH, drug content, and entrapment efficiency of GTX-NLC (formulation F2) over three months of storage at 4, 25, and 40 °C (mean ± SD, n = 3).

The lead NLC colloidal dispersion (F2) was analyzed at scheduled time intervals for any changes in PS, PDI, ZP, pH, assay, and EE (%) on storage (Figure 4).

Overall, the formulation exhibited a good stability profile across the three storage conditions tested. There was no visual evidence of cracking, expulsion, change in color, or drug sedimentation from the nanocarrier lipid matrix. Kalam et al. observed progressive crystallization and solidification of the lipids in the formulated SLNs at 20 °C, causing a decrease in EE from 89.2% to 85.1% over a span of 3 months [31]. In our studies, the assay and EE values of the lead F2 formulation were 99.6 ± 2.3% and 97.0 ± 1.3%, including the samples stored at 40 °C, indicating good chemical stability. There were no statistically significant (*p* < 0.05) differences in the PS, PDI, ZP, and pH values, even after 90 days, indicating excellent physical stability. Therefore, it can be concluded that the lead NLC formulation (F2) was stable at all tested storage conditions.

### 3.11. Antibacterial Activity Testing

Antibacterial studies were conducted to determine the antimicrobial efficiency of the lead formulation against GTX-C. F2 was chosen as the lead formulation over F6, as it had a desired release profile while maintaining similar physiochemical characteristics. The antimicrobial efficiency was evaluated against the two commonly isolated leading pathogens in ocular infections, MRSA and *P. aeruginosa.* Post-operative and nosocomial ocular infections due to MRSA have also been observed, with *P. aeruginosa* being the most frequent isolate among Gram-negatives [4]. In-house antibacterial studies showed a MIC of 6.25 µg/mL against MRSA and *P. aeruginosa* for both the lead formulation as well as GTX-C (Table 6). Moreover, this experimental MIC was achieved at all release time points samples during the study (0.5 to 24 h). The antibacterial activity is due to the GTX present in the formulations, as the placebos did not show any such activity.

### 3.12. STEM

**Figure 5:**
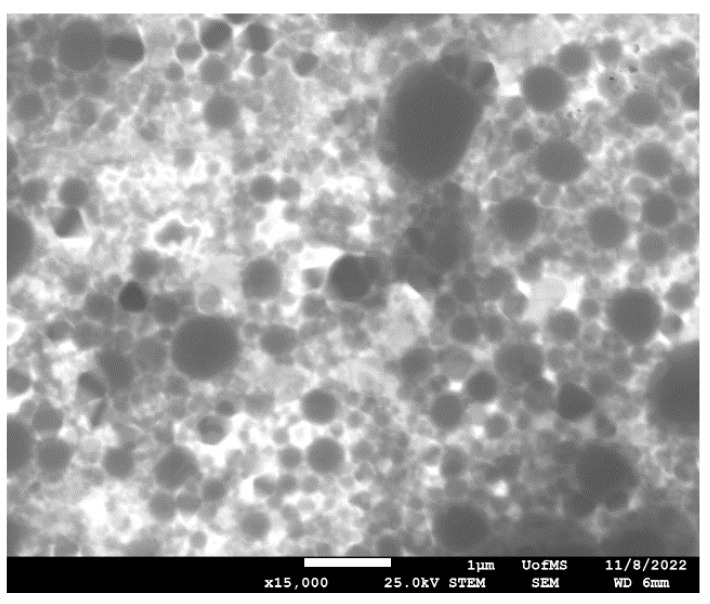
Scanning transmission electron microscopic image of F2 at 15 k magnification.

The STEM images of the lead F2 formulation confirmed the presence of (spherical) lipid droplets, as shown in Figure 5.

## 4. Conclusions

GTX-NLC formulations were successfully prepared and characterized. The lead NLC (F2) was physiochemically stable for over three months at the three tested storage conditions. The formulation showed an extended GTX release profile, over a 12 h period, and demonstrated similar antibacterial activity as the GTX-C eyedrops. Moreover, the MIC_90_ against the tested microorganism was attained with the very first time-point tested. Ex vivo transcorneal permeation studies showed significant improvement in GTX permeation from the GTX-NLC formulation, compared to the commercial ophthalmic solution eyedrops. Overall, the GTX-NLC formulation developed in the current investigation could reduce the frequency of dosing, by improving treatment outcomes, thereby increasing patient compliance compared to commercial GTX eyedrops. Future in vivo research could reveal the difference in ocular surface residence time and biodistribution after topical administration. In conclusion, the NLC formulations could provide an efficient GTX delivery platform for the management of conjunctivitis as well as various other ocular bacterial infections.

## Figures and Tables

**Table 1:** Lipid screening studies for gatifloxacin-loaded lipid nano formulations.

Liquid Oil	Solubility	Solid Lipids	Solubility
Castor oil	(−)	Precirol^®^ ATO 5	(+)
Sesame oil	(−)	Compritol^®^ 888	(−)
Oleic acid	(+)	Dynasan^™^ 114	(−)
Mineral oil	(−)	Geleol^™^	(+)
Capryol^®^ 90	(−)	Gelucire^™^ 43/01	(−)
Labrafac^®^ Lipophile WL 1349	(−)	Dynasan^™^ 116	(−)
Isopropyl Myristate, NF	(−)	Gelucire^™^ 50/13	(−)
Olive oil	(−)	Gelucire^™^ 44/1	(−)
Miglyol^®^ 829	(−)	Softisan 154	(−)

(+): GTX dissolves in the lipid and does not precipitate after cooling to RT. (−): GTX does not dissolve in the lipid, or dissolves in the lipid but precipitates on cooling.

**Table 2:** Composition of placebo lipid nano formulations.

Placebo	Formulation	Precirol^®^ ATO 5 (% *w*/*v*)	Geleol™ (% *w*/*v*)	Oleic Acid (% *w*/*v*)	Visual Examination
NLCs	P1	3.0	-	2.0	NLCs were formed
	P2	2.5	-	2.5	NLCs were formed
	P3	3.0	-	1.0	NLCs were formed
	P4	2.0	-	2.0	NLCs were formed
	P5	-	3.0	2.0	Phase separation during the addition of the aqueous phase
	P6	-	2.5	2.5	Phase separation during the addition of the aqueous phase
	P7	-	3.0	1.0	Phase separation during the addition of the aqueous phase
	P8	-	2.0	2.0	Phase separation during the addition of the aqueous phase
SLNs	P9	5.0	-	-	SLNs were formed
	P10	4.0	-	-	SLNs were formed
	P11	-	5.0	-	Phase separation during the addition of the aqueous phase
	P12	-	4.0	-	Phase separation during the addition of the aqueous phase

Note: aqueous phase contains Tween^®^ 80 (3% *w*/*v*), Glycerin (2.25% *w*/*v*), TPGS (0.002% *w*/*v*), and Milli-Q water (q.s. to 10 mL) for all nanocarriers.

**Table 3:** Physicochemical characteristics and visual observations gatifloxacin loaded lipid nano formulations (mean ± SD, n = 3).

Formulation *		Precirol^®^ ATO 5 (% *w*/*v*)	Oleic Acid (% *w*/*v*)	Physicochemical Characteristics						Visual Observations
				PS (nm)	PDI	ZP (mV)	pH	Assay (%)	EE (%)	
NLCs	F1	3.0	2.0	266.8 ± 6.4	0.42 ± 0.05	−32.2 ± 0.5	6.37 ± 0.03	99.2 ± 2.6	87.3 ± 2.0	Milky-white dispersion
	F2	2.5	2.5	216.2 ± 6.8	0.32 ± 0.03	−30.6 ± 1.2	6.37 ± 0.03	98.6 ± 2.1	91.63 ± 1.5	Milky-white dispersion
	F3	3.0	1.0	229.5 ± 3.2	0.16 ± 0.06	−26.3 ±0.9	5.62 ± 0.03	100.5 ± 2.4	73.9 ± 3.2	Milky-white dispersion
	F4	2.0	2.0	209.2 ± 3.2	0.14 ± 0.03	−31.2 ± 1.3	6.24 ± 0.02	102.3 ± 1.1	81.2 ± 1.8	Milky-white dispersion
SLNs	F9	5.0	-	200 ± 11.0	0.20 ± 0.03	−21.3 ± 0.4	7.17 ± 0.03	101.0 ± 2.5	80.29 ± 2.3	Drug expulsion at day 15 with 20.3 ± 2.1% EE
	F10	4.0	-	182.7 ± 4.3	0.15 ± 0.02	−18.2 ± 0.6	7.12 ± 0.02	97.9 ± 3.1	71.7 ± 2.8	Drug expulsion at day 15 with 12.2 ± 3.6% EE

* All formulations contained GTX (0.5% *w*/*v*). Note: aqueous phase contains Tween^®^ 80 (3% *w*/*v*), Glycerin (2.25% *w*/*v*), TPGS (0.002% *w*/*v*), and Milli-Q water (q.s. to 10 mL) for all nanocarriers.

**Table 4:** Effect of Tween^®^ 80 concentration on particle size, polydispersity index, zeta potential, drug content, and entrapment efficiency of gatifloxacin-loaded nanostructured lipid carrier formulations (mean ± SD, n = 3).

Formulations *		Tween^®^ 80 (% *w*/*v*)	Physicochemical Characteristics						Visual Observations
			PS (nm)	PDI	ZP (mV)	pH	Assay (%)	EE (%)	
NLCs	F2 ^#^	3.0	216.2 ± 6.8	0.32 ± 0.03	−30.6 ± 1.2	6.37 ± 0.03	98.6 ± 2.1	90.4 ± 1.5	Milky-white dispersion
	F5 ^#^	2.0	239.5 ± 7.1	0.38 ± 0.04	−29.3 ± 0.5	6.42 ± 0.01	99.5 ± 2.6	70.6 ± 2.5	Milky-white dispersion
	F6 ^#^	4.0	200.3 ± 2.4	0.18 ± 0.02	−30.2 ± 0.3	6.39 ± 0.01	100.2 ± 2.2	96.9 ± 2.0	Milky-white dispersion
	F4 ^@^	3.0	209.2 ± 3.2	0.14 ± 0.03	−31.2 ± 1.3	6.24 ± 0.02	102.3 ± 1.1	83.1 ± 1.8	Milky-white dispersion
	F7 ^@^	2.0	226.4 ± 5.4	0.25 ± 0.03	−29.4 ± 0.7	6.44 ± 0.01	98.7 ± 4.0	77.5 ± 2.1	Milky-white dispersion
	F8 ^@^	4.0	183.4 ± 5.6	0.13 ± 0.02	−28.9 ± 1.0	6.35 ± 0.01	99.3 ± 3.1	87.1 ± 2.9	Milky-white dispersion

* All formulations contained GTX (0.5% *w*/*v*). ^#^ Total lipids (5% *w*/*v*); Precirol^®^ ATO 5: oleic acid (1:1). ^@^ Total lipids (4% *w*/*v*); Precirol^®^ ATO 5: oleic acid (1:1).

**Table 5:** Mathematical model fitting for release kinetics of gatifloxacin-loaded nanostructured lipid carrier formulations (mean ± SD, n = 3).

Formulation	Korsmeyer–Peppas	Higuchi	First-Order	
	R^2^			n
F2	0.9999	0.9094	0.9895	0.6
F6	0.9976	0.9515	0.982	0.6

Korsmeyer–Peppas model: log% drug released vs. log time, Higuchi model: % drug released vs. square root of time; first-order model: amount of drug remaining vs. time; zero-order is not relevant, hence excluded from the table.

**Table 6:** MIC_90_ values for the control, F2, and placebo formulations against methicillin-resistant *Staphylococcus aureus* and *Pseudomonas aeruginosa*.

Formulation	MIC_90_ (μg/mL)	
	Methicillin-Resistant *Staphylococcus aureus*	*Pseudomonas aeruginosa*
GTX-C	6.25	6.25
F2	6.25	6.25
Placebo F2	Not achieved	Not achieved

F2—gatifloxacin loaded nanostructured lipid carrier formulation.

## Data Availability

The data presented in this study are available upon request from the corresponding author.

## Supplementary Materials

The following supporting information can be downloaded at: https://www.mdpi.com/article/10.3390/antibiotics12081318/s1, Table S1. HPLC chromatographic conditions for gatifloxacin.
