# Supplementary material for: Gatifloxacin Loaded Nano Lipid Carriers for the Management of Bacterial Conjunctivitis
Source: Antibiotics (Basel). 2023 Aug 15;12(8):1318. doi: 10.3390/antibiotics12081318 (PMC10451836; doi:10.3390/antibiotics12081318)
Supplement: Supplementary file 1 [file antibiotics-12-01318-s001.zip › antibiotics-2512653-supplementary.pdf]

Gatifloxacin Loaded Nano Lipid Carriers for the management of Bacterial Conjunctivitis

Table S1. HPLC chromatographic conditions for gatifloxacin.

Solvent delivery module	Alliance Waters e2695 separations module
Data processor	Waters® Empower Chromatography Data System Software
Sensitivity	2.0 AUFS
Mobile phase	A mixture of phosphate buffer (18 mM) containing 0.1% v/v triethylamine (pH 2.8, adjusted with dilute phosphoric acid) and methanol (60:40 v/v)
Column	Waters Symmetry® C_{18}, (150 x 4.6 mm, 5 µm)
Flow rate	1.2 mL/min
Injection volume	20 µL
Detector	UV-Vis
Wavelength (λ max)	294 nm
Column temperature	Room temperature
Sample holder	Room temperature
Retention time	6.5 min
Linearity range	1-100 µg/mL (R^2 = 0.998)
Limit of detection	0.24 µg/mL
Limit of quantification	0.71 µg/mL
Accuracy	% recovery was within 100.8-102 using 9 determinations over 3 concentration levels covering the specified range (10, 20, and 80 µg/mL) with % RSD < 2.0
% RSD for Precision	< 2
